# Hypovitaminosis D and risk factors in pediatric epilepsy children

**DOI:** 10.1186/s12887-021-02906-7

**Published:** 2021-10-02

**Authors:** Napakjira Likasitthananon, Charcrin Nabangchang, Thitiwan Simasathien, Suchavadee Vichutavate, Voraluck Phatarakijnirund, Piradee Suwanpakdee

**Affiliations:** 1 Neurology Division, Department of Pediatrics, Phramongkutklao Hospital, Bangkok, Thailand; 2 Endocrine Division, Department of Pediatrics, Phramongkutklao Hospital, Bangkok, Thailand

**Keywords:** Pediatric epilepsy; vitamin D deficiency, Hypovitaminosis D, Anti-seizure medications

## Abstract

**Background:**

Anti-seizure medication (ASM) treatment is one of the significant risk factors associated with abnormal vitamin D status in epilepsy patients. Multiple studies have shown that adult epilepsy patients can exhibit vitamin D deficiency. However, there are few reports investigating pediatric epilepsy patients. In this study, we aimed to identify risk factors related to hypovitaminosis D in pediatric epilepsy patients in Thailand.

**Methods:**

A cross-sectional retrospective cohort study was conducted in 138 pediatric epilepsy patients who received anticonvulsants from April 2018 to January 2019. Demographic data, seizure types, puberty status, physical activity, duration, and types of anti-seizure medications were analyzed. Patients with abnormal liver function, abnormal renal function, and who received vitamin D supplements or ketogenic diet containing vitamin D were excluded. Levels of serum vitamin D (25(OH)D) were measured.

**Results:**

All 138 subjects were enrolled, the age ranged from 1.04 – 19.96 years; (mean = 9.65 ± 5.09), the mean serum 25(OH) D level was 26.56 ± 9.67 ng/ml. The prevalence of vitamin D deficiency was 23.2% and insufficiency was 47.8% respectively. Two risk factors—puberty status (OR 5.43, 95% CI 1.879-15.67) and non-enzyme-inhibiting ASMs therapy (OR 3.58, 95% CI 1.117-11.46)—were significantly associated with hypovitaminosis D, as shown by multivariate analyses.

**Conclusions:**

Our study reports the high prevalence of hypovitaminosis D in pediatric epilepsy patients in Thailand despite being located in the tropical zone. These findings can guide clinicians to measure vitamin D status in pediatric epilepsy patients particularly when they reach puberty and/or are using non-enzyme-inhibiting ASMs therapy. Early detection of vitamin D status and prompt vitamin D supplementation can prevent fractures and osteoporosis later in life.

**Trial registration:**

TCTR20210215005 (http://www.clinicaltrials.in.th/).

## Background

Epilepsy is one of the most common neurological disorders, globally as well as in Thailand. The prevalence rate of epilepsy in Thailand was approximately 7.2 per 1000 population [1]. Anti-seizure medications (ASMs) are the mainstay of treatment. The drugs have various effect and adverse effect profiles, as well as mechanisms of actions. Among other things, they cause cognitive impairment [2].

Additionally, vitamin D deficiency is another known adverse effect of ASMs [3] Essentially, 90% of the vitamin D in the human body is synthesized in the skin following exposure to sunlight, particularly to ultraviolet B with a wavelength of 209–305 nm, which is most abundant in sunlight between 10 am and 2 pm. Substrates in the skin are then converted to vitamin D_3_ (cholecalciferol). Foods such as cod liver oil, milk fat, butter, animal liver, and egg yolk are another source of vitamin D, in the form of vitamin D_2_ (ergocalciferol). Both vitamin D_3_ and vitamin D_2_ are metabolized in the liver and converted by 25-hydroxylases into calcidiol (25[OH]D), a prehormone that is the major circulating form of vitamin D and is used to determine an individual’s vitamin status. Circulating 25(OH) D is eventually metabolized in the kidneys to a more biologically active form known as calcitriol (1,25(OH)_2_D), which serves various functions such as increasing calcium and phosphorus absorption in the intestines, inhibiting the secretion of parathyroid hormone, and modulating the formation and development of bones and teeth [4].

Anti-seizure medications can reduce vitamin D levels, increase parathyroid hormone levels, and cause hypocalcemia and hypophosphatemia, resulting in an increased risk of bone fracture in patients with long-term ASM use [5]. In addition, studies have found that ASMs increase vitamin D destruction in the liver by stimulating the activity of the liver enzyme cytochrome (CY)P450 [6].

The prevalence rate of vitamin D deficiency in epileptic patients in the United States is reportedly 11.9% in adults and 25% in children aged between 3 and 17 years [7, 8]. Comparably, it is 22.5% in children with epilepsy aged between 3 and 18 years in Malaysia [9]. A similar prevalence (23.3%) has been found among children with epilepsy in Thailand [10].

In previous studies, risk factors for vitamin D deficiency are found to be female sex, adolescents over 12 years of age, high BMI, developmental delay and limited physical activity, enzyme-inducing ASMs, polytherapy, using drugs over a long period of time, Indian race, and exposure to sunshine less than 60 min per day [7–10].

However, there are few reports of the prevalence of hypovitaminosis D, including vitamin D insufficiency and deficiency, in pediatric epilepsy patients treated with different ASMs. We aim to determine the prevalence and risk factors of hypovitaminosis D in children with epilepsy.

## Methods

This study was designed as both a cross-sectional study to evaluate the prevalence of vitamin D deficiency and a retrospective cohort study to investigate the factors affecting vitamin D levels in children with epilepsy who were treated with ASMs. Ethical approval was obtained from the Institutional Review Board of the Royal Thai Army Medical Department (R227h/60). Informed consent was obtained from the patients or their parents/guardians.

We enrolled 138 pediatric epilepsy patients aged 1–20 years at the Neurology pediatric clinic at Phramongkutklao Hospital from April 2018 to January 2019. Patients that had abnormal kidney or liver function, received vitamin D supplements, or were on a ketogenic diet containing vitamin D were excluded.

The data collected from the patients included age, gender, weight, height, BMI, types of seizures, and cause of epilepsy. Puberty status was assessed using Tanner staging. Daily activity abilities were divided into two categories; normal activity and limited activity, such as walking with aid, using a wheelchair, or bedridden. Developmental status was initially evaluated using the Denver II test. If developmental delay was suspected, patients under 6 years of age were assessed with Mullen Scales of Early Learning (MSEL), and the children older than 6 years of age were tested with the Wechsler Intelligence Scale for Children-Third Edition. The duration of sun exposure between 10 am and 2 pm per day were assessed through an interview and recorded.

The name, number, and duration of the ASM treatments were recorded. Participants were divided into the enzyme-inducing ASM (carbamazepine, phenobarbital, and phenytoin), valproate, and benzodiazepine (BZD) and newer ASMs (diazepam, clonazepam, clobazam, topiramate, levetiracetam, lamotrigine, zonisamide, vigabatrin, oxcarbamazepine, gabapentin, lacosamide, perampanel, and rufinamide) groups based on the type of ASM used.

Serum 25(OH) D levels were used to categorize the vitamin D status, which was defined as vitamin D sufficiency: 25(OH) D level >  30 ng/ml (75 nmol/L), vitamin D insufficiency: 25(OH) D level 20–30 ng/ml (50–75 nmol/L), and vitamin D deficiency. 25(OH) D level < 20 ng/ml (75 nmol/L) [ 11 ] . In this study, we combine the insufficiency and deficiency groups into the hypovitaminosis D group for the purpose of our analyses.

Statistical analyses were performed using IBM SPSS Statistics 25.0 for Mac (IBM Corporation, Armonk, NY). Fisher’s exact and the chi-square test were used to analyze categorical data. The Mann–Whitney U test was used to analyze continuous data. Logistic Regression was used to predict risk factors of hypovitaminosis D. A *p*-value < 0.05 was considered statistically significant.

## Results

### Vitamin D status

The prevalence of vitamin D deficiency was 23.2% (*n* = 32) and that of Vitamin D insufficiency was 47.8% (*n* = 66). Thus, 71% (*n* = 98) of the patients had hypovitaminosis D.

### Demographic characteristics

**Fig. 1:**
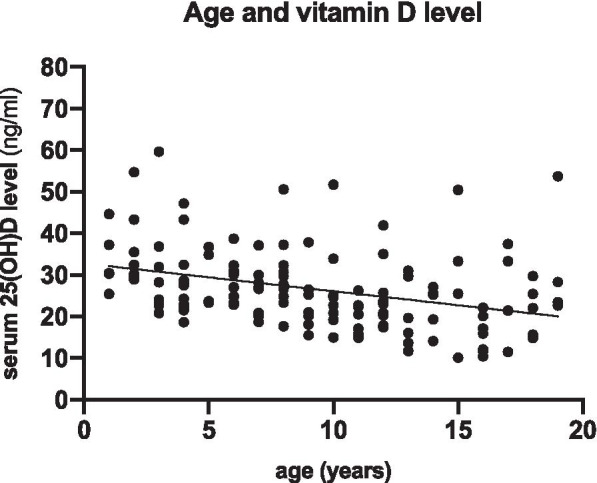
The data of age and serum vitamin D (25(OH)D) level showed almost the patients of all age were serum vitamin D less than 30 ng/ml, related with the prevalence of hypovitaminosis D 71%. The older age trend to decrease the level of serum vitamin D

The demographic characteristics of all 138 pediatric epilepsy patients are shown in Table 1. The mean age of the participants was 9.65 ± 5.09 years (range: 1.04–19.96 years). The mean serum 25(OH) D level was 26.56 ± 9.67 ng/ml. The relationship between age and vitamin D level is shown in Fig. 1. Most patients, irrespective of age, had a serum 25(OH) D level less than 30 ng/ml, and lower levels were associated with a higher age.

**Table 1:** Demographic data

Risk factor	Total subjects ***n*** = 138	Vitamin D sufficiency 25(OH)D > 30 ng/ml ***n*** = 40	Hypovitaminosis D 25(OH)D ≤ 30 ng/ml ***n*** = 98	***P***-value
**Age**				
(years)	9.65 ± 5.09	6.8 ± 5	10.21 ± 4.69	< 0.001*
**Serum 25(OH) D level**				
(ng/mL)	26.56 ± 9.67	38.3 ± 8.18	21.77 ± 5.03	< 0.001*
**Gender**				
male	74(53.6%)	26(35.1%)	48(64.9%)	0.087
female	64(46.4%)	14(21.9%)	50(78.1%)	
**BMI** (Body mass index)				
(kg/m^2^)	17.69 ± 4.82	16.94 ± 4.59	18.01 ± 4.91	0.239
**Type of seizure**				
focal	131(94.9%)	39(29.8%)	92(70.2%)	0.379
generalize	7(5.1%)	1(14.3%)	6(85.7%)	
**Cause of epilepsy**				
remote symptomatic cause	52(37.7%)	14(26.9%)	38(73.1%)	0.888
genetic cause	12(8.7%)	4(33.3%)	8(66.7%)	
idiopathic cause	74(53.6%)	22(29.7%)	52(70.3%)	
**Puberty status**				
prepuberty	81(58.7%)	33(40.7%)	48(59.3%)	< 0.001
puberty	57(41.3%)	7(12.3%)	50(87.7%)	
**Physical status**				
normal daily activities	102(73.9%)	27(26.5%)	75(73.5%)	0.273
limited daily activities	36(26.1%)	13(36.1%)	23(63.9%)	
**Gross motor development**				
normal	69(50%)	16(23.2%)	53(76.8%)	0.133
delay	69(50%)	24(34.8%)	45(65.2%)	
**Fine motor development**				
normal	67(48.6%)	15(22.4%)	52(77.6%)	0.097
delay	71(51.4%)	25(35.2%)	46(64.8%)	
**Language development**				
normal	61(44.2%)	14(23%)	47(77%)	0.164
delay	77(55.8%)	26(33.8%)	51(66.2%)	
**Personal-social development**				
normal	60(43.5%)	14(23.3%)	46(76.7%)	0.199
delay	78(56.5%)	26(33.3%)	52(66.7%)	
**Duration of sun exposed, during 10 am-14 pm**				
< 60 min/day	129(93.5%)	35(27.1%)	94(72.9%)	0.121
≥ 60 min/day	9(6.5%)	5(55.6%)	4(44.4%)	

*p-value significant at < 0.05

Table 2 shows the types of ASM used, which includes enzyme-inducing ASMs (26 patients) such as carbamazepine (15 patients), phenobarbital (6 patients), and phenytoin (6 patients). One patient used both phenobarbital and phenytoin. The enzyme-inhibiting ASM group, which only included valproate, was comprised of 53 patients, whereas the BZD and newer ASMs group comprised 113 patients. The most commonly used newer ASM was levetiracetam (80 patients), followed by topiramate (53 patients). Seventeen and 36 patients received a low (less than 5 mg/kg/day) and high (5 mg/kg/day or more) dose of topiramate, respectively. Other ASMs in this group were benzodiazepines such as clonazepam, diazepam, and clobazam (21 patients); lamotrigine (5 patients); zonisamide (2 patients); vigabatrin (3 patients); lacosamide (10 patients); perampanel (11 patients); and rufinamide (1 patient).

**Table 2:** ASMs characteristics and vitamin D status

ASMs characteristics	Total subjects n = 138	Vitamin D sufficiency 25(OH)D > 30 ng/ml n = 40	Hypovitaminosis D 25(OH)D ≤ 30 ng/ml n = 98	***P***-value
**Monotherapy**				
no	71(51.4%)	24(33.8%)	47(66.2%)	0.201
yes	67(48.6%)	16(23.9%)	51(76.1%)	
**Duration of ASMs used**				
≤ 2 years	33(23.9%)	15(45.4%)	18(54.5%)	0.017*
> 2 years	105(76.1%)	25(23.8%)	80(76.2%)	
**Enzyme-inducer ASMs** (CBZ,PB,PHT)				
no	112(81.1%)	34(30.4%)	78(69.6%)	0.461
yes	26(18.9%)	6(23.1%)	20(76.9%)	
**Enzyme-inhibitor ASMs** (VPA)				
no	85(61.6%)	20(23.5%)	65(76.5%)	0.074
yes	53(38.4%)	20(37.7%)	33(62.3%)	
**BZD and Newer ASMs**				
no	25(18.1%)	6(24%)	19(76%)	0.545
yes	113(81.9%)	34(30.1%)	79(69.9%)	
**Levetiracetam**				
no	58(42.1%)	16(27.6%)	42(72.4%)	0.758
yes	80(57.9%)	24(30%)	56(70%)	
**Topiramate**				
no	85(61.6%)	26(30.6%)	59(69.4%)	0.599
yes	53(38.4%)	14(26.4%)	39(73.6%)	
**Topiramate dosage**				
< 5 mg/kg/day	17(32.1%)	7(41.2%)	10(58.8%)	0.092
≥ 5 mg/kg/day	36(67.9%)	7(19.4%)	29(80.6%)	

*ASMs* anti-seizure medications; *CBZ* Carbamazepine; *PB* Phenobarbital; *PHT* Phenytoin; *VPA* Valproate; *BZD* Benzodiazepine

*p-value significant at < 0.05

### 3. Factors predicting vitamin D status (Table 3)

Univariate logistic regression analyses revealed that the potential risk factors of hypovitaminosis D were puberty and ASM use of more than 2 years, with a 4.9- and 2.7-times increased risk of hypovitaminosis D, respectively (*p* <  0.05).

**Table 3:** Univariate and multivariate logistic regression analysis evaluated risk factors predicting hypovitaminosis D

Factor	Univariate model			Multivariate model		
	Crude Odds Ratio	95%CI	p-value	Adjusted Odds Ratio	95%CI	p-value
**Gender**						
male	1			1		
female	1.935	0.904-4.14	0.089	1.568	0.649-3.786	0.318
**Puberty status**						
prepuberty	1			1		
puberty	4.911	1.983-12.159	0.001*	5.426	1.879-15.67	0.002*
**Duration of sun exposed, during 10 am-14 pm**						
< 60 min/day	3.357	0.852-13.223	0.083	2.68	0.455-15.79	0.276
≥ 60 min/day	1			1		
**Monotherapy**						
No	1			1		
Yes	1.628	0.772-3.434	0.201	1.49	0.442-5.028	0.520
**Enzyme-inducer ASMs** (CBZ,PB,PHT)						
No	1			1		
Yes	1.453	0.536-3.939	0.463	1.306	0.372-4.582	0.677
**Enzyme-inhibitor ASMs** (VPA)						
No	1.97	0.932-4.162	0.074	3.578	1.117-11.46	0.032*
Yes	1			1		
**BZD and Newer ASMs**						
No	1.363	0.500-3.712	0.545	2.029	0.376-10.941	0.411
Yes	1			1		
**Duration of ASMs used**						
≤ 2 years	1			1		
> 2 years	2.667	1.175-6.05	0.019*	2.222	0.827-5.969	0.113

*ASMs* anti-seizure medications; *CBZ* Carbamazepine; *PB* Phenobarbital; *PHT* Phenytoin; *VPA* Valproate; *BZD* Benzodiazepine

*p-value significant at < 0.05

Multivariate logistic regression analysis adjusted for other risk factors such as gender, puberty status, duration of sun exposure, monotherapy drug, enzyme-inducing ASMs, enzyme-inhibiting ASMs, newer ASMs, and more than 2 years of ASM use suggested that puberty status and ASM type were significantly related to hypovitaminosis D. Specifically, patients who had undergone puberty had a 5.4-times (95% confidence interval 1.9–15.7) higher risk of hypovitaminosis D than pre-pubescent patients. Patients who had received non-enzyme-inhibiting ASMs had a 3.5-times (95% confidence interval 1.1–11.5) higher risk of hypovitaminosis D than those who received enzyme-inhibiting ASMs (valproate).

## Discussion

Which role the drugs play for the vitamin D deficiency is still uncertain [7–9, 11, 12]. VPA seems to exert the least influence, while the effect of the new generation of ASMs one cannot say anything certain about, based on the results from this study.

We found that the prevalence rate of vitamin D deficiency in pediatric epilepsy patients in Bangkok, Thailand was 23.2%, which was similar to the rates reported in previous studies conducted in Thailand [10] and Malaysia [9]. However, when we considered both vitamin D deficiency and vitamin D insufficiency together as hypovitaminosis D, our results indicated that approximately two-thirds of pediatric epilepsy patients had hypovitaminosis D, even though Thailand is located in the tropical zone.

Similar to the previous reports, we also found puberty status to be a key risk factor of hypovitaminosis D. This may be because puberty represents a critical period of skeletal bone mineralization, which results in a high rate of vitamin D utilization and increased need for vitamin D, leading to high rates of deficiency [13].

The other factors included in our analyses, namely BMI, cause of epilepsy, physical activity status, developmental status, ASMs used for monotherapy or polytherapy, and duration of sun exposure, were not significantly associated with hypovitaminosis D.

More than 2 years of ASM use was significantly associated with hypovitaminosis D in the univariate analysis but not in the multivariate analysis. This may be because various drugs were simultaneously studied, and they may affect vitamin D levels through different mechanisms.

Interestingly, 53 of the 138 patients (38%) were receiving an enzyme-inhibiting ASM (valproate). The multivariate analysis revealed that those treated with non-enzyme-inhibiting ASMs had a 3.5-times higher risk of hypovitaminosis D than those treated with enzyme-inhibiting ASMs. This finding is similar to those of previous studies that found normal levels of active vitamin D in patients receiving valproate therapy [14–16]. One study found that patients receiving valproate monotherapy had fewer negative effects on bone markers and better bone density than those receiving enzyme-inducing ASM (phenytoin, carbamazepine) monotherapy [17]. However, the findings from studies of bone metabolism in patients receiving valproate are controversial, and it is difficult to make a comparison because of varying study designs [18]. More evidence is needed on the effect of valproate use on bone health.

Among our participants, the most commonly used newer ASMs were levetiracetam (57.9%) and topiramate (38.4%). As seen in previous studies, we found no statistically significant association between newer ASM use and vitamin D level. This may be because newer ASMs, except for topiramate and oxcarbazepine, do not affect CYP450, the enzyme that regulates the metabolism of vitamin D in the liver [19, 20]. Additionally, another study showed no association between the use of newer ASMs and decreased bone mineral density [21].

**Fig. 2:**
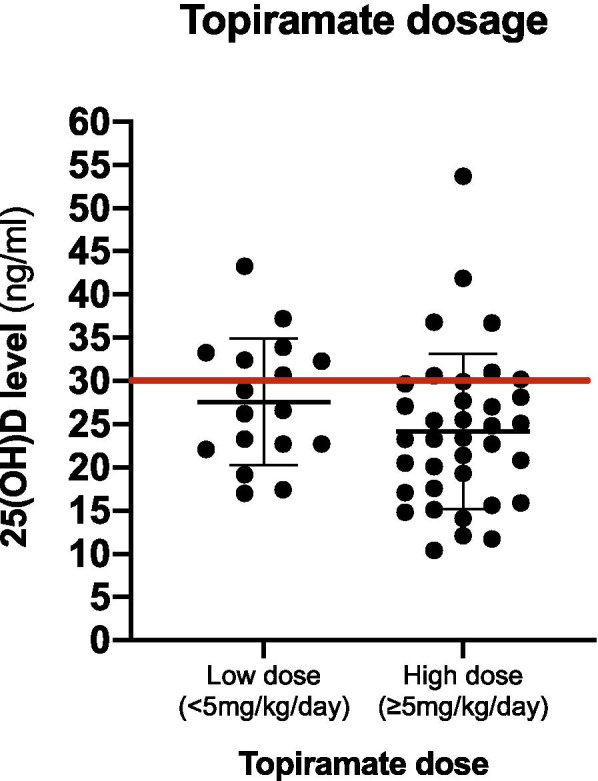
Topiramate was compared between 2 groups, one was a low dose (< 5 mg/kg/day) in 17 patients and the other was a high dose (≥ 5 mg/kg/day) in 36 patients. The result was showed the high dose group was more hypovitaminosis D (25(OH) D < 30 ng/ml) than the low dose group, 80.6, and 58.8% respectively

Among newer ASMs, topiramate has unique pharmacokinetics in that it acts as an enzyme-inhibiting agent (metabolized via CYP2C19 in the liver) at low doses (< 200 mg/day in adults) and as an enzyme-inducing agent (metabolized via CYP3A4) at high doses (≥ 200 mg/day) [19, 20, 22]. Hence, we divided participants receiving topiramate into the low dose (< 5 mg/kg/day) and high dose (≥ 5 mg/kg/day) groups in our analysis. We found that more patients in the high dose group had hypovitaminosis D than in the low dose group (80.6 and 58.8%, respectively), although this difference was not statistically significant (*p* = 0.092, Fig. 2).

We recognize that our study has a limitation of a small sample size. A multicenter study with a large number of participants should be conducted in the future to confirm our results.

## Conclusion

Our study shows a relatively high prevalence rate of vitamin D deficiency and vitamin D insufficiency of 23.2 and 47.8%, respectively, even though Thailand is located in the tropical zone. The key risk factors of hypovitaminosis D are puberty status and non-enzyme-inhibiting ASM use. There is currently a lack of guidelines on the appropriate time to assess the vitamin D status of epilepsy patients. Our findings suggest that clinicians who treat pediatric epilepsy patients, especially those entering puberty and being prescribed non-enzyme-inhibiting ASMs should consider regular monitoring of vitamin D adequacy. In cases of hypovitaminosis D, clinicians should recommend prompt vitamin D supplementation as it is essential to prevent pathological fractures and osteoporosis for the patients in later life.

## Data Availability

The dataset analysed during the current study is available from the corresponding author to researchers on reasonable request.

## Abbreviations

1,25(OH)_2_D: Calcitriol
25(OH)D: Calcidiol
ASMs: Anti-seizure medications
BMI: Body mass index
BZD: Benzodiazepine
CBZ: Carbamazepine
PB: Phenobarbital
PHT: Phenytoin
VPA: Valproate
